# Ceramic Composite Materials Obtained by Electron-Beam Physical Vapor Deposition Used as Thermal Barriers in the Aerospace Industry

**DOI:** 10.3390/nano10020370

**Published:** 2020-02-20

**Authors:** Bogdan Stefan Vasile, Alexandra Catalina Birca, Vasile Adrian Surdu, Ionela Andreea Neacsu, Adrian Ionut Nicoară

**Affiliations:** 1 National Research Center for Micro and Nanomaterials, University Politehnica of Bucharest, 010164 Bucharest, Romania; alexandra.birca@upb.ro (A.C.B.); ionela.neacsu@upb.ro (I.A.N.);; 2 Department of Science and Engineering of Oxide Materials and nanomaterials, Faculty of Applied Chemistry and Materials Science, University Politehnica of Bucharest, 010164 Bucharest, Romania; 3 National Research Centre for Food Safety, University Politehnica of Bucharest, 010164 Bucharest, Romania

**Keywords:** thermal protection systems, ultrahigh temperature applications, EB-PVD

## Abstract

This paper is focused on the basic properties of ceramic composite materials used as thermal barrier coatings in the aerospace industry like SiC, ZrC, ZrB_2_ etc., and summarizes some principal properties for thermal barrier coatings. Although the aerospace industry is mainly based on metallic materials, a more attractive approach is represented by ceramic materials that are often more resistant to corrosion, oxidation and wear having at the same time suitable thermal properties. It is known that the space environment presents extreme conditions that challenge aerospace scientists, but simultaneously, presents opportunities to produce materials that behave almost ideally in this environment. Used even today, metal-matrix composites (MMCs) have been developed since the beginning of the space era due to their high specific stiffness and low thermal expansion coefficient. These types of composites possess properties such as high-temperature resistance and high strength, and those potential benefits led to the use of MMCs for supreme space system requirements in the late 1980s. Electron beam physical vapor deposition (EB-PVD) is the technology that helps to obtain the composite materials that ultimately have optimal properties for the space environment, and ceramics that broadly meet the requirements for the space industry can be silicon carbide that has been developed as a standard material very quickly, possessing many advantages. One of the most promising ceramics for ultrahigh temperature applications could be zirconium carbide (ZrC) because of its remarkable properties and the competence to form unwilling oxide scales at high temperatures, but at the same time it is known that no material can have all the ideal properties. Another promising material in coating for components used for ultra-high temperature applications as thermal protection systems is zirconium diboride (ZrB_2_), due to its high melting point, high thermal conductivities, and relatively low density. Some composite ceramic materials like carbon–carbon fiber reinforced SiC, SiC-SiC, ZrC-SiC, ZrB_2_-SiC, etc., possessing low thermal conductivities have been used as thermal barrier coating (TBC) materials to increase turbine inlet temperatures since the 1960s. With increasing engine efficiency, they can reduce metal surface temperatures and prolong the lifetime of the hot sections of aero-engines and land-based turbines.

## 1. Introduction

One branch of engineering that deals with the maintenance, development and study of airplanes and spacecraft is aerospace engineering, where research into materials for the construction of aerospace components is in continuous development. Although metals are the most widely used materials in aircraft components, discoveries in materials science, particularly in composite science and technology, have allowed the development of new materials for aerospace engineering [1,2]. Lightweight design of aircraft frames and engines with materials of improved mechanical properties can improve fuel efficiency, increase payload, and flight range, which directly reduce the aircraft operating cost [3,4,5].

The aerospace industry is based on the use of composite materials for both primary and secondary constitutional components such as engine nacelles, rocket motor castings, aircraft wings, antenna dishes, landing gear doors, centre wing boxes, tall cones, engine cowls and others [6,7].

At the present time, the use of composite materials in the aerospace industry inspire in a positive way the development and outline of modern and complex aero vehicles. In this sense, the properties like high specific strength and individual stiffness together with other unique properties makes this type of materials very attractive and suitable for this kind of applications. A class of composite materials is classified as advanced composites which is defined by metal matrix composites, high-performance fibre-reinforced polymers, and those most used in high-performance aerospace vehicles, and their properties are the ceramic matrix composites. This class of composite materials provide supplementary functional advantages, the most highlighted being the temperature resistance [8,9]. Using composite materials in developing parts of aero vehicles implies more than just replacing the metals or other regular materials, it is about the introduction of advanced materials which have a role in a multitude of features starting from new designs in morphological structures, which were initially not possible with traditional materials [10].

One of the problems in the development of some aero vehicles consists in obtaining parts that must have specific properties for the field of use. The most attractive characteristic of advanced composite materials is based on the high ratio between strength, which is a basic feature when speaking about aerospace, and weight which is another goal in this industry, compared to the metals frequently used in aerospace. Moreover, the production techniques are a very important subject in this field. Manufacturing components by using composite materials favors the production of numerous distinct structures [11,12].

When it comes to temperatures that can be reached in this field, the aerospace industry has an ultrahigh temperature class that is generally placed from 1600 °C and can reach up to 2200 °C [13]. These temperatures require the use of materials that can withstand very high temperatures and also have exclusive mechanical properties [14,15].

Considering that a single material cannot have as many properties as are needed for aerospace applications, there has been a need to study and develop composite materials that have advantages that situate them in an advantageous position when it comes to their use in the aerospace industry. The latest air vehicles models contain more than 50% of their weight in terms of composite materials. However, there is still a lack of information regarding mechanical behavior, which leads to stricter regulations to guarantee safety standards [3,16]. This has led to the impossibility of reaching the full potential of the composites in the aerospace industry and, of course, to the need for further studies [11].

**Figure 1:**
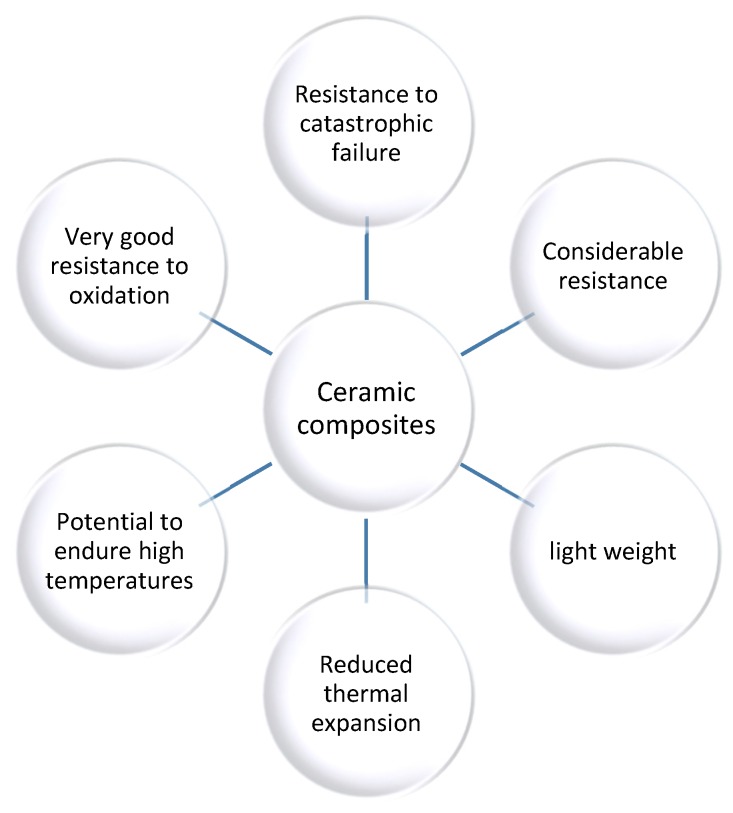
Properties of ceramic composites [18].

**Figure 2:**
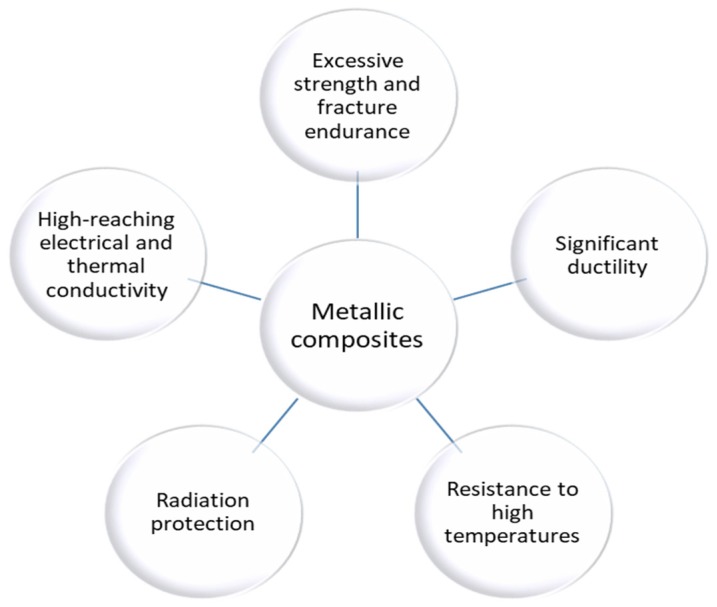
Properties of metallic composites [18].

Ceramic composites are obtained by linking ceramics using continuous fibers, particles or whiskers. The literature data provide information about the conventional types of reinforcement for ceramic matrix composites which include silicon carbide, titanium carbide and boron carbide, silicon nitride and boron nitride, alumina and zirconia, carbon and boron. Below are presented the advantageous characteristics of ceramic composites (Figure 1) [1,17]:

Metallic composites are manufactured by reinforcing various types of metal matrices, such as titanium, aluminum, copper, magnesium, etc. [19]. Typical blends for metal composites are ceramic particle or fiber in particular, but carbon fiber or metallic fiber can also be used. When it comes to processing techniques, metal composites can be obtained by diverse methods such as casting and powder metallurgy, but with specific limitations because of the metallic use [20]. This is despite the fact that There are limitations in the aerospace industry for metallic composites, the properties of which are presented in the Figure 2 [1]:

Another class of materials that have applications in the aerospace industry is represented by the ultra-high temperature ceramics. These materials are described as possessing a blend of properties that are characterized by very good and suitable mechanical properties and at the same time a significant meting point, which can reach up to 3000 °C and even exceed this value [13,21]. 

In this sense, the materials that possess specific thermo-mechanical and thermo-chemical properties are required for aerospace applications, especially in ultra-high temperature area [22,23]. The ultra-high temperature class includes several applications like the manufacturing of solid rocket motors which need a very high temperature that are increased starting from room temperature to approximatively 3000 °C. Because of the fact that the application takes place at high temperatures, it is necessary that the materials for the components such as rocket combustion chambers to have properties that are dependent on each other such as high melting temperature, high-reach strength and of course significant resistance to environmental factors. Hypersonic vehicles also require components part manufactured from materials that reflect properties that ensure a specific action at temperatures beyond 1600 °C [24]. 

Some of the most notable properties of the materials that have applications at high temperatures are good oxidation resistance, high melting point, high hardness, and thermal shock and ablation endurance [14].

Demonstrating the behavior of different materials under high temperature applications, it was concluded that these materials should present a layer or more that covers the surface of the materials. At this point in time, thermal barrier coatings represent a subject that involves numerous and modern deposition techniques to increase the properties of the usual materials that are used in developing the component parts of aero vehicles. Surfaces of engines and gas turbine blades are the most covered components for the reason that at high temperature there is a need for thermal barrier behavior, considering the action of this as thermal insulation to the high temperature gas that flows within the turbine blades [25]. By covering the surface of aero vehicle components with materials that act as a thermal barrier, this also leads to a reduction in the thermal stresses. Criteria of thermal barrier coatings are to present low weight and low thermal conductivity, but there is still an issue because of the fact that after the heat-treatment processes, thermal conductivity of the coatings may increase [26].

## 2. Thermal Barrier Coating 

Thermal-barrier coatings are defined as ceramic materials that present suitable resistance at high temperatures. Components like metal turbine blades used in aircraft engines need to be covered by depositing thermal barrier which allow these engines to perform at high temperatures [27]. The activity of these coatings is based on protecting from oxidation or melting because of that fact that hot gases from the engine core may affect the metal that is used at manufacturing these components for aero vehicles [25].

One essential role of thermal-barrier coatings components is to present various properties against the harsh environment such as corrosive atmosphere, high temperature and variation of this and complex stress conditions. It is well understood that it is complicated for a single coating component to possess all these conditions. At this level of depositing the coatings, the thermal barrier layers are planned to last for thousands of landings and take-offs in aero engines. When speaking about the complexity and diversity of thermal barrier coatings structures, there is an impediment of premature failure that can appear during operating conditions [28]. At one point in time, the use of thermal barrier coatings decreased and moreover, their full characteristics were discredited. In order to avoid and eliminate these impediments, more detailed analyses were considered regarding materials, processing principles, performance and, not least, failure mechanisms were enhanced, in order to better understand how to respond beneficially. This research field presents associative subjects of materials science, chemistry, physics, mechanics and thermodynamics [29]. 

At the same time, the advantageous development of thermal barrier coatings are essential to bring improvements in the case of inlet gas temperature which leads to a boost of the performance of gas turbines. Hence, to develop thermal barrier coatings with interdependent features such as high resistance to sintering, low thermal conductivity and also phase stability, it is necessary to highlight the increased demands in order to obtain a proper final material [30]. Commonly, thermal barrier coatings include a ceramic top coat and a metallic bond coat. The utilization of a bond coat is required to secure the metal substrate in the case of oxidation and corrosion because of the high temperature and also for coupling the ceramic top coat and the metallic substrate, being located between the substrate and the ceramic top coat [31]. 

However, work has been published on the conventional thermal-barrier coatings system, which in fact contain three layers, covering the substrate. The first layer is the metallic bond coat, the second layer is the middle thermally grown oxide and the third layer is the ceramic top coat. Separately, these layers cannot provide the thermal and mechanical properties necessary for their use under special conditions, but which are directly proportional to the processing conditions that may impose modifications [32,33].

The first layer seems to possess critical characteristics, due to the fact that this layer performs two fundamental roles. The certainty of the coatings system starts with the first layer, in this sense, one role is to ensure a very good adhesion between the substrate and the ceramic top layer. The second function is to act in the case of severe oxidation, because the oxygen ions from the environmental conditions may pass through the ceramic layer, due to the porosity and high diffusivity. The top coat requires high thermal stability and low thermal conductivity [34].

For these layers to act as demanded under special conditions at high temperatures, it is necessary that them to become common parts with the metal substrate that need coatings. For this reason, diverse physical methods were developed to deposit the ceramic top coat as a thermal barrier coating to the metallic substrate. The following methods are electron beam physical vapor deposition (EB–PVD), laser chemical vapor deposition, and atmospheric plasma sprayed, high-velocity oxy-fuel, sol-gel, plasma spray physical vapor deposition [1,34,35]. One of the most used of these kinds of application is electron beam physical vapor deposition (EB-PVD) and the second is atmospheric plasma spray (APS) [27,31,36].

Over time, measures have been taken to improve the competency of a gas turbine. These actions leaded to operating temperatures exceeding 1300 °C, which require thicker thermal barrier coating which influence the chemistry together with an additional cooling system. As a result, the top coat layer, present an increase of thickness which manage the surface temperature of the thermal barrier coating to a faster cooling components system with a rate of temperatures of 4–9 °C along with 25 µm [32].

The research in this domain surrounded by experimental activity and the implication of numerous people concluded that that thermal barrier coatings must meet a number of well-defined and interdependent conditions. The first condition speaking about aero vehicles is to present low weight, also low thermal conductivity is required. Because of the fact that the environmental medium may suffer drastic thermic changes, the coatings should resist variation from heating to cooling and vice versa and indeed to thermal shock. In order not to encounter problems that can have a significant impact later, the coatings must be chemically compatible with the substrate and resist oxidation process [32,37]. Thermal insulation is another mandatory condition for thermal-barrier coatings to the elemental superalloy engine components. The compliance of the superalloy parts with the thermal expansion is another necessity to minimize the discrepancy stresses. Moreover, thermal-barrier coatings must reverse as much as possible of the radiant heat produced by hot gas and is mandatory to prevent the contact of the heat with the substrate. It is desired for the thermal barrier coatings to ensure thermal protection for the coated substrate and to be capable of resisting for prolonged service times [31].

How to improve the protection of the components that are in contact with high temperatures, which use thermal-barrier coatings, has attracted the attention of researchers for many years. The coatings are deposited on the substrate using, in general, EB-PVD methods. This advanced technique involves high electron beam heating of rough materials which subsequently generate steam. The produced steam will be subjected to the substrate surface which is deposited as a coating [38]. It is understood that the coating is formed as a layer of vertical column grains that are standing upright on the substrate. Between the columns there are consecutive gaps, that separate pores in the structure of the grains which can be open pores or closed pores. Due to these structures of the coatings, the characteristics of the thermal barrier will be improved [39].

## 3. Electron Beam Physical Vapour Deposition (EB-PVD) Technology

The EB-PVD method is based mostly on the activity of the electron beam, which is considered the most important part having a role as thermal source in this deposition technique. One of the best and most attractive features of EB-PVD is the capability of depositing all types of material. The deposition procedure is based on the action of an electron beam established at 2000 °C within an electron gun, acting in accordance with the acceleration of thermal electrons supported by high voltage. The equipment includes a target of the material of interest, which is subsequently hit by high-speed electrons. Due to the energy generated by the electrons, the target material is melted and after that the material is transformed into vapor and deposited on the surface of the substrate as a coating. The highlighted advantage of this technique is the high deposition rate compared to other coating technique. The parameters applied for specific materials can be managed more easily and the surface also can be controlled when speaking about the dimension of the deposition. One mandatory property in obtaining the deposition materials is to present a strong adhesion between the coating and the substrate, which in the case of use of the EB-PVD technique, is fulfilled [29,31,40].

**Figure 3:**
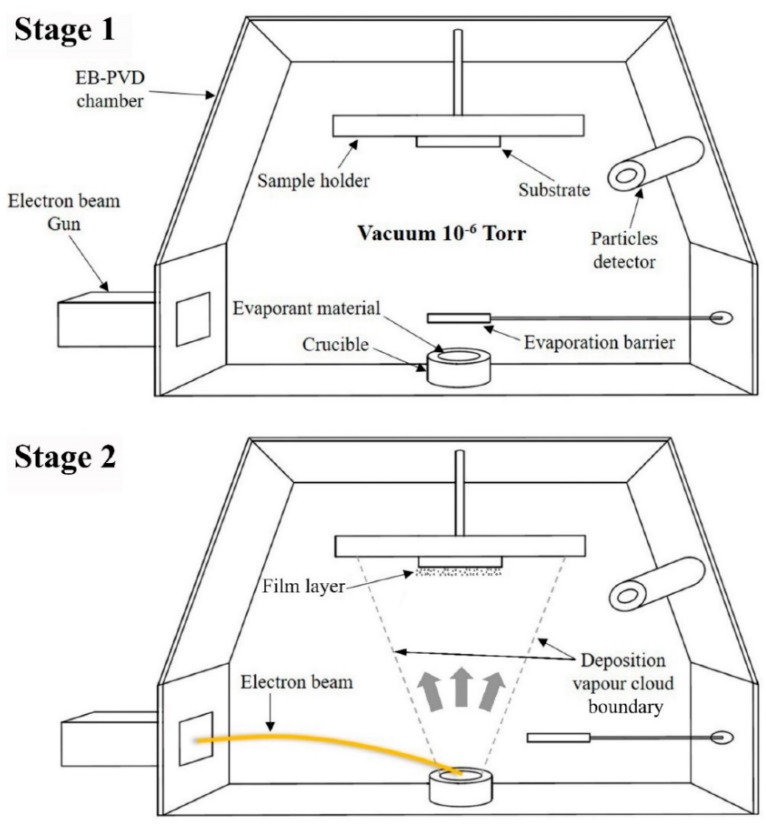
Schematic of electron beam physical vapor deposition (EB-PVD) equipment (Stage 1) and the generation of the film for coating [43].

Depending on the needs of the final material, the coatings can be deposited differently from ceramic to ceramic, metallic to metallic, ceramic to metallic, or metallic to ceramic. Moreover, the best of the characteristic of this deposition technique is the multi material that can be used. In this sense, multilayer coatings can be deposited and also may be disposed of like alternative layers of distinct composition comprising ceramics, metals and polymers. All of these materials can be arranged as different and various layers on the substrate. Pointing to time efficiency, in this technique the deposition rate is high, and also in a short period the coating presents a dense structure. The microstructure may be controlled surrounded by a managed composition, trying to erase every possibility to be contaminated, and all of these properties are obtained finally regarding easily controlled parameters and flexible deposition. There are only minor exceptions where the deposited layers do not have a homogeneous microstructure, but generally the finished materials possess a good surface and uniform microstructure. Therefore, there is a fine relationship between the manipulating the process parameters and the final microstructure of the materials and also uniformity [39,41,42]. Below are showed the schematic illustrations of electron beam physical vapor deposition (EB-PVD) equipment (Stage 1) and the generation of the film for coating (Figure 3).

## 4. Ultra-High Temperature Ceramics

Over time there has been new materials and modifications of the materials in the aerospace field have been developed. A new generation of aero vehicles are based on the incorporation of components that are composed from a special class of materials known as ultra-high temperature ceramics. These kind of materials are used as thermal protection and in the engine parts of the space vehicles, and in fact ultra-high temperature ceramics can be also used in critical applications on the ground where is a need of resistance to high temperature [44].

Ultra-high temperature ceramics appear in the periodic table in the groups IVB and VB transition metals, and are based especially on carbides along with nitrides and borides. These ceramics exhibit a superior combination of properties characterized by high melting points together with mechanical properties. In this sense, the use of ultra-high temperature ceramics in extreme environments make them excellent potential candidate for these applications [13].

Extreme applications require the use of materials that are not susceptible to oxidation attack in particular, and by using single-phase materials excluding secondary phases materials is not enough. The single phase materials own all the undesired properties for the use in extreme environment such as low thermal shock resistance, low fracture toughness which make these kinds of material unacceptable for aero vehicle applications and also for engineering parts of the vehicles. To erase all the possibilities of failure, the best way is to use a combination of at least two secondary phase of ultrahigh temperature ceramics. One of the most used composites contain silicon carbides (SiC) or other ceramics that involve silicon in different microstructures such as particles, whiskers or fibers. By using composite materials with the required special properties for aerospace application a better thermal shock resistance will be displayed in aggressive environments [45,46].

Ultra-high temperature applications proposed after years of testing and research, and most used with high potential materials in extreme environments, are fundamentally substances such as C (carbon), Ta (tantalum), W (wolfram), Os (osmium), Re (rhenium) and non-oxide compounds such as monocarbides, diborides and mononitrides of transition metals of IVB and VB groups in the periodic table, highlighted as Ti (titanium), Hf (hafnium), Zr (zirconium), Nb (niobium), and Ta (tantalum) [47].

Research interest in aluminum matrix composites has also increased in the last few years, referring to aerospace industries based on the properties of these, such as low density and high strength. From the various types of materials, Al_2_O_3_ is the most usual ceramic, forming a composite matrix by reinforcing with others materials [48,49]. Alumina have constantly been considered proper for aerospace applications both at ambient and at elevated temperatures. Even if the Al_2_O_3_ possess polymorphs character, the corundum α-Al_2_O_3_ is found to be the most suitable form for applications which include a medium temperature. However, oxide ceramics are ideal candidates when speaking about the high-temperature applications due to the fact that these ceramics possess proper behavior in oxidative environments and characteristic high melting point, but especially in combination with a material that supports these properties [50].

In this sense, a material which provides a better view in the aerospace application by forming a composite matrix with Al_2_O_3_ is tantalum carbide TaC. Its melting point is 3997 °C and it possesses the greatest chemical stability among other carbide [51]. Moreover, the properties of the TaC such as low thermal expansion and high electrical conductivity stimulate the use of this attractive candidate to establish a composite material with aluminum matrix. The literature data provide information about the difficulty developing a composite material by reinforcing TaC particles, but at the smallest possible size. Also the distribution of these particles represents an issue in the development process, because of the fact that the normal distribution of the smallest TaC particles in the alumina matrix is very hard to obtain. The agglomeration process occurs when it is desire to distribute the smallest particles in alumina matrix [52]. Off all the reinforcement particles in the matrix processes, the powder metallurgy process supports in the best way the uniform distribution of the particles in the matrix, however, there are also some problems with the agglomeration mechanism in this process. In this case, when the particles present agglomeration and the composite materials are assesed for sintering, there are possibilities to appear and to retain porosities, which can lead to unappropriated mechanical properties [53,54]. From the sintering method point of view, a spark plasma sintering process based on using aluminum matrix composites has proper behavior when developing adequate dense composites which also possess suitable mechanical properties, in comparison to conventional sintering methods [55,56].

As a basic idea, from the chemical point of view, all ultra-high temperature ceramics are compounds of carbon, boron, or nitrogen in combination with at least one of the early transition metals of IVB and VB in the periodic table. The binary compounds (transition metals and carbon, boron or nitrogen) finally present strong covalent bonds leading to properties of a composite material such as high melting temperature, high stiffness and high hardness. Moreover, all of the characteristics of the ultra-high temperature ceramics are increased compared to oxide ceramics. Due to the fact that ultra-high temperature ceramics involve the action of a mix of ceramics and metals, the final features make the materials suitable and attractive for extreme temperatures and other aggressive conditions of the application environment highlghting capabilities that are beyond other materials [57].

## 5. Ceramic Matrix Composites

Aerospace engineering includes an important part which is based on the choice of the materials for aero vehicles components. The requirements for a material vary simultaneously and in direct correlation with the specific component that possesses a suitable property for the aerospace industry. Some particular behaviors are being in consideration in materials selections when the design of a vehicles is desired. Each component is analyzed for design requirements which consist in manufacturability, loading conditions, maintainability and geometric limits. Aircraft engines are a point of interest in engineering this component. The most important aspects are the weight reduction and thrust improvement, which mandatory implicate materials with superior properties. The engine materials should present some specific features such as low densities which leads to weight reduction, and it is very important to possess essential mechanical properties under high-temperature conditions and an aggressive oxidative environment. Speaking about the design of an aircraft turbine engines, two divisions are described. The cold sections consist of the compressor, fan and casting, and the hot sections consisting of chamber, combustion and turbine. The category of cold and hot suggest that the sections present different temperatures, which affect the material selection where temperature is a crucial condition for aircraft engine materials. Corrosion resistant and high specific strength materials are suitable for use in the cold section. Composites that include titanium or aluminum and polymers are optimal materials for the cold section. The temperature that is reached in this section is usually in the range of 500–600 °C. On the other hand, for the hot section the materials should present high temperature resistance, hot corrosion resistance and high specific strength. In this section, the temperature is usually between 1400–1500 °C. Titanium composite in this section can not be used, in this case the suitable materials are nickel superalloys, due to their significant high temperature resistance strength [3,7,10,58,59].

The use of composite materials in aerospace vehicle engineering is about more than putting together the individual properties and increasing the final composite material characteristics and behavior. By means of using composite materials, the weight is reduced and the assembly is less complex. Moreover, the use of composite materials involves reducing fuel burn which is a major problem, and also reducing greenhouse gas emissions. Two methods can help to accomplish reduction in fuel burn. The first is about redicing the weight of gas turbine engines, and the second is about raising the thermal performance of the engines. As a matter of fact, composite materials are involved in both situations [2].

Even if the developing stages of composite materials compared to the developing stages of metal production seem to be identical, at the final stage the properties will be specific and beyond the classic metal product and manipulate the design procedures for composite systems. During the process of designing a composite material, at each step various options are available, making the design process persistent and interactive. The design of a component part from an aero vehicle, such as an airframe or a wing involve a considerable number of design variables. These variables need to accomplish various constraints from particular disciplines and also diverse targets have to be performed. Relevant models are used to correlate the constraints and targets to the design variables. At this point in time, aircraft designers possess the ability to use new techniques consisting in multidisciplinary design optimization. Due to the reason that high-performance computational tools are now available, changes and modifications can be correlated at every step of the design process under desired conditions [10,60,61].

The very varied options accessible in the nature and category of matrix and reinforcing components generate composite materials with a broad variety of pattern and characteristics which are an interdependent association of the particular constituent features [1,62].

### 5.1. Carbon–Carbon Composites

Carbon–carbon composite materials are part of a category of materials that are called advanced composite materials, due to their properties. A large variety of shapes are characteristic to this type of materials starting from one-dimensional to *n*-dimensional (usually *n* = 1,2 or maximum 3), conditioned by the raw utilized material. By taking into account this benefit, the performance of the materials can be customized in direct contact with the applications. The first use of carbon–carbon composites was in the aerospace domain of applications; at the present time, this type of composites possesses various properties with applications in numerous sectors that brings them to the fore of research into ceramic composite materials [59,63].

For aerospace applications, carbon-based ceramic composites possess attractive properties, such as remarkable thermal stability and also low weight, making them the most favorable materials. Carbon fibers and the carbon matrix are basically components of engineered carbon–carbon composite materials, occasionally improved with different components. One attractive characteristic is the selection of the constituent materials and fiber orientations, which highlight the possibility to manage the properties of the final carbon–carbon composites. Generally, carbon–carbon materials and components are created at the same time, so that the final composite properties can be directed to increase the component capabilities. Resistance to oxidation at high temperatures, fracture toughness, strength and stiffness are principal characteristics of this composite carbon materials [59,64].

This blend of characteristics, leads to their use as preferred materials for manufacturing numerous aero vehicles components parts such as landing gear door, flaps, ailerons and others. Still, the deficiency of stability above 500°C in aggressive environments has placed them in the category of materials that require enhancing. Because of this major drawback, only for short duration can they be used in a harsh environment. However, these composites can endure very high heat fluxes, but only for limited durations, which makes them appropriate for parts of the vehicles that not require continuous withstand for long durations such as re-entry nose tips. Furthermore, the carbon–carbon composites can be improved by extending the application duration and multiple consecutive use. There are some methods to improve the oxidation resistance such as coatings with a material exhibiting oxidation resistance. The second method is to enhance the composite matrix by supplementing with a third phase or to modify the carbon matrix to carbides such as silicon carbide (SiC). By improving the oxidation resistance with the addition of Si, carbon fibre-reinforced SiC matrix composites, are termed C/SiC composites. The oxidation and erosion resistance is enhanced due to the properties of the C/SiC composites. Additionally, the C/SiC composites can be used for lightweight and harsh applications, due to the fact that the density of the carbon is below the density of numerous metallic materials [65,66,67].

### 5.2. Hafnium Carbide (HfC) Composites

Pointing to one of the most important properties in the aerospace applications, hafnium carbide (HfC) present the maximal melting point (∼3950 °C) among the transition metal carbides. Another attractive feature is low vapour pressure, good ablation resistance and chemical inertness [68,69].

Some recent publications reveal a new experience by introducing HfC compounds towards carbon–carbon composites. Wang et al. described the possibility of obtaining a hafnium carbide coating for carbon–carbon composite substrate by using the chemical vapor deposition method [70], and another coating for carbon–carbon composites by co-deposition of hafnium (tantalum) carbon using the same chemical vapor deposition technique [71]. A different method was reported by Li et al. where the deposition of hafnium carbide on the carbon–carbon composites was possible by immersing the carbon materials in a hafnium oxychloride aqueous solution [72]. To offer protection for carbon–carbon composites, hafnium and silicon carbide multilayers were deposited under low-pressure chemical vapour deposition as coatings [73].

The high environmental temperature of aero applications has significant action upon the materials. Some tests were performed to evaluate the strengths of HfC ceramics at different temperature. In this sense, from room temperature to up to ~ 2200 °C a strength of approximatively 350 MPa was recorded, which declines with the increase of the temperature. At 2200 °C plastic deformation appeared, as a result of grain-boundary sliding. This test highlights the essential role of grain boundaries, because in HfC ceramics with smaller grain size, the decline was more considerable [14,74].

### 5.3. Carbon/Silicon Carbide (C/SiC) Composites

Among the ceramic materials, silicon carbide (SiC) is placed as a first choice when a high-temperature environment is present. This material is used especially for structural components of aerospace vehicles such as transportation and nuclear areas, due to the fact that SiC possesses significant thermal conductivity, remarkable specific strength and superior tribology behavior at raised temperatures. Like any other material, it also has properties that do not meet the necessary conditions, such as low fracture resistance which limits in some cases the utilization of it in applications of interest. In this sense, given the subject discussed above (see the 5.1 carbon–carbon composites subsection), the carbon fiber-reinforced silicon carbine ceramic matrix composite materials are seeming to fulfill the requirements for high temperature applications. The fracture resistance is upgraded, and also the strength is increased with the supplement of high strength fibers [14,75].

The addition of carbon fiber in the silicon carbide ceramic matrix, increase the final composite material characteristics, highlighting noticeable material properties such as high strength, superior thermal shock resistance surrounded by good oxidation resistance, low density and a specific feature of managing and maintaining the mechanical properties even if the applications are under elevated temperatures. All of these properties determine the material to use in extreme conditions including oxidizing atmosphere, as manufacturing materials for components of aero vehicles. It is well known that by obtaining a composite material the final structure will be improved together with the characteristics. A better oxidation resistance is manifested in carbon–carbon SiC composites, compared to individual materials. Due to the fact that on the surface of the substrate, the silica offers a protective layer, the behavior under oxidizing atmosphere of the composite is improved. Moreover, light weight is a property that is more accentuated in the composite material compared to the individual one, and also the economic part is ameliorated because the carbon matrix is easier to develop than silicon carbide matrix [76,77].

Another way in maintaining a suitable activity of the materials is to incorporate silicon carbide fiber in the silicon carbide matrix. The components of the aero vehicles like gas turbine engines offer the best options when its manufacture includes the utilization of silicon carbide fiber reinforced silicon carbide. To evaluate the stress rupture properties, the high temperature composite materials which consist of, basically, SiC, were investigated under 100 MPa as a moderate stress level. The results of SiC–SiC composite showed it to be able to operate at temperature beyond 1315°C. Carbon–carbon composite and carbon fiber-silicon carbide exhibited advantageous and preferred stress rupture properties at elevated temperature. Moreover, SiC–SiC composites, results with an advanced in the durability of the resistance at oxidation atmosphere, compared to carbon–carbon and carbon fiber–silicon carbide [2,77,78].

### 5.4. Zirconium Carbide/Silicon Carbide (ZrC/SiC) Composites

Ultra-high temperature applications include the utilization of zirconium carbide (ZrC), as one of the best options due to the fact that the exceptional properties performed with suitable activity of the ZrC under harsh conditions. At high temperatures, the ZrC composite generate a refractory oxide scale which is another advantage when it comes to oxidation [14].

Transition metal carbides have considerable properties, being in the focus of the researchers for manufacturing aero vehicles components with required properties such as high melting point, high hardness and chemical stability, which are characteristics for zirconium carbide. Moreover, ZrC possesses features like impressive hardness which is mandatory for many cutting tools or/and abrasive industries. Numerous papers, place the zirconium carbide as a suitable material for elevated temperature applications due to high corrosion resistance [79].

Rocket engine nozzles and hypersonic vehicles components during their applications, are in direct contact with aggressive environment. For this reason, the materials used in manufacturing these components have to present firstly a high melting temperature. Zirconium carbide ceramic is a promising material in this way. However, there are in this case some limits of the materials such as poor sinterability because of the fact that ZrC possesses a reduced self-diffusion coefficient and strong covalent bonding. By a poor sinterability is understood the fact that it is more complicated to reach a completely dense composites without a support from sintering additives. Because of the fact that ZrC ceramic composites may have limits in terms of their full activity under special conditions having poor thermal shock resistance and low fracture toughness, by adding SiC into ZrC the properties may be improved. The mechanical properties and oxidation resistance of ZrC are clearly enhanced after the incorporation of SiC, leading to the generation of a melted SiO_2_ layer at high temperature and also to the discrepancy of thermal expansion coefficient among ZrC and SiC [80,81].

### 5.5. Zirconium Diboride/Silicon Carbide (ZrB_2_/SiC) Composites

Another excellent candidate for applications at high temperatures is zirconium diboride (ZrB_2_). This diboride is similar to zirconium carbide having attractive properties such as low density, high melting point, remarkable chemical inertness, and it is used as thermal protection barrier on the substrate of aerospace vehicles. However, in this case too the individual zirconium diboride did not reach all the required conditions because has some inconvenience such as low fracture toughness and low oxidation resistance. Moreover, it is a similarity between zirconium diboride and zirconium carbine when it comes to manufacturing completely dense samples. This process is limited by the undesirable characteristics of ZrB_2_ such as strong covalent bond and reduced self-diffusion coefficient, and because of the impurities on the surface of substrate materials. Also in this case, the addition of SiC brings a benefic difference, changing the properties and increasing the mechanical properties, the thermal and oxidation resistance. In the same time, the exaggerated grain growth of zirconium diboride is avoided with the addition on silicon carbide [82].

### 5.6. Aluminum Oxide/Zirconium Dioxide (Al_2_O_3_/ZrO_2_) Composites and Zirconium Dioxide/Silicon Dioxide (ZrO_2_/SiO_2_) Aerogels

An attractive oxide ceramic candidate for aerospace application is ZrO_2_. The characteristics of this ceramic are represented by a very high melting point at a temperature of ~2700 °C, promising mechanical properties and stability in oxidative conditions [83]. The use of ZrO_2_ as thermal barrier coatings has been a favorable choice for several years and even in the present time is still recommended. A difference between the traditional ZrO_2_ coatings and nanostructured ZrO_2_ coatings may have a large influence on the properties of the final material. The research data reveal that the nano structure of ZrO_2_ has improved the properties of the material with higher toughness, lower thermal conductivity, higher bonding strength, and higher wear resistance [84]. Over the years, there has been interest regarding the use of zirconia as fully stabilized zirconia, partially stabilized zirconia and tetragonal zirconia polygonal [83].

To obtain a better performance in a special high temperature environment, the involvement of the scientific community has focused on the use of the Al_2_O_3_/ZrO_2_ eutectic ceramic as a thermal barrier coating. This composite is obtained as a melt growth composite material, meaning of a eutectic reaction between the matrix phase and the second phase which occurs when oxide melt is solidifying. The final composite is made by Al_2_O_3_ and ZrO_2_ being developed at the same time and together, which can possess a micro or nano structure. Due to the fact that, Al_2_O_3_/ZrO_2_ eutectic ceramics possess excellent behavior at high temperatures such as oxidation resistance and high temperature strength became a new generation of materials used as thermal barrier coatings and may even overcome the properties of SiC at high temperatures [85,86,87].

Another new generation of composite materials is based on ZrO_2_/SiO_2_ aerogel. Silica aerogel is produced using nanoparticles as aggregate and form a three-dimensional structure by interconnecting each nanoparticle between them. However, the silica aerogels can resist only at a temperature below 600 °C if there is a need for a long working conditions [88]. In this sense, the composite of ZrO_2_/SiO_2_ aerogel have improved results under high temperature due to ultra-low thermal conductivity. Additionally, this composite presents more outstanding properties such as low density and a better heat insulation leading to a thermal stability at a temperature of 1000 °C [89,90]. However, there are some issues speaking about the mechanical strength and fragility of aerogels. Some augmented methods have been used in order to obtain a better result, such as including ceramic fibers or functional polymers (epoxy, polyurethane and polyethylene) by cross-linking them with the aerogels. As a comparison between organic or inorganic reinforcement, it has been demonstrated that the inorganic reinforcement is obvious and confirmed as having potential due to its supportive stability behavior at high temperature [91].

Due to the fact that the materials used in aerospace applications must have a suitable behavior at high temperatures, below are some properties that are taken into account when choosing these materials, based on those discussed in this review (Table 1). 

## 6. Conclusions

Thermal-barrier coatings obtained by using the electron beam physical vapour deposition technique represent a way to improve the behavior of aero vehicles in high-temperature applications. The coatings have a significant role in assuring a barrier which acts in high-temperature environments. The performance of the thermal barrier is enhanced thanks to various ceramic coats deposited on the substrate. 

In order to choose suitable materials for aerospace applications, it has been proven that ceramic materials have properties that are mandatory for such applications. Ceramic materials possess low thermal conductivities and, for this reason, it is desirable for the manufacturing of components for aero vehicles to contain a large proportion of ceramic composites. The performance of the engine is increased, the temperature of the metal substrate is reduced and managed, and the lifetimes of the engines, hot sections and turbines are prolonged only by covering with thermal-barrier coatings. 

The selection of materials for acting as a thermal barrier is based on the evaluation of the materials. Basic requirements are mandatory such as high melting point, low thermal conductivity, chemical inertness, good adherence to the metallic substrate, high-temperature resistance, high strength and resistance to oxidation at high temperatures. However, until now, no single material can achieve all of these mandatory conditions. 

## Figures and Tables

**Table 1:** Melting temperature and mechanical properties of various materials used as thermal barrier coatings.

Material	Melting Temperature (°C)	Hardness (GPa)	Young’s Modulus (GPa)
TaC	3427 [92]	20.6 ± 1.2 [92]	579 ± 20 [92]
HfC	3890 [93]	31.5 ± 1.3 [92]	552 ± 15 [92]
SiC	2730 [93]	25.5 [94]	450 [95]
ZrC	3530 [96]	31.3 ± 1.4 [97]	507 ± 16 [97]
ZrB_2_	3245 [98]	21 [99]	490 [99]
ZrO_2_	2699 [100]	11.77 [101]	171 [102]
Al_2_O_3_	2071 [100]	21.58 [101]	380 [102]
